# Occurrence of *Salmonella enterica* and *Escherichia coli* in raw chicken and beef meat in northern Egypt and dissemination of their antibiotic resistance markers

**DOI:** 10.1186/s13099-017-0206-9

**Published:** 2017-10-18

**Authors:** Amira A. Moawad, Helmut Hotzel, Omnia Awad, Herbert Tomaso, Heinrich Neubauer, Hafez M. Hafez, Hosny El-Adawy

**Affiliations:** 1 Friedrich-Loeffler-Institut, Institute of Bacterial Infections and Zoonoses, Naumburger Str. 96a, 07743 Jena, Germany; 2 Bacteriology department, Animal Health Research Institute (AHRI), Mansoura branch, Mansoura, 35516, Egypt; 3 Faculty of Veterinary Medicine, Kafrelsheikh University, Kafr El-Sheikh, 33516 Egypt; 4 Faculty of Veterinary Medicine, Mansoura University, Mansoura, Egypt; 5 Institute of Poultry Diseases, Free University Berlin, Berlin, Germany

**Keywords:** *Salmonella*, *Escherichia coli*, Antibiotic resistance, Integron, Egypt

## Abstract

**Background:**

The global incidence of foodborne infections and antibiotic resistance is recently increased and considered of public health concern. Currently, scarcely information is available on foodborne infections and ESBL associated with poultry and beef meat in Egypt.

**Methods:**

In total, 180 chicken and beef meat samples as well as internal organs were collected from different districts in northern Egypt. The samples were investigated for the prevalence and antibiotic resistance of *Salmonella enterica* serovars and *Escherichia coli*. All isolates were investigated for harbouring class 1 and class 2 integrons.

**Results:**

Out of 180 investigated samples 15 *S. enterica* (8.3%) and 21 *E. coli* (11.7%) were isolated and identified. *S. enterica* isolates were typed as 9 *S.* Typhimurium (60.0%), 3 *S.* Paratyphi A (20.0%), 2 *S*. Enteritidis (13.3%) and 1 *S.* Kentucky (6.7%). Twenty-one *E. coli* isolates were serotyped into O1, O18, O20, O78, O103, O119, O126, O145, O146 and O158. The phenotypic antibiotic resistance profiles of *S. enterica* serovars to ampicillin, cefotaxime, cefpodoxime, trimethoprim/sulphamethoxazole and tetracycline were 86.7, 80.0, 60.0, 53.3 and 40.0%, respectively. Isolated *E. coli* were resistant to tetracycline (80.9%), ampicillin (71.4%), streptomycin, trimethoprim/sulphamethoxazole (61.9% for each) and cefotaxime (33.3%). The dissemination of genes coding for ESBL and AmpC β-lactamase in *S. enterica* isolates included *bla*_CTX-M_ (73.3%), *bla*_TEM_ (73.3%) and *bla*_CMY_ (13.3%). In *E. coli* isolates *bla*_TEM_, *bla*_CTX-M_ and *bla*_OXA_ were identified in 52.4, 42.9 and 14.3%, respectively. The plasmid-mediated quinolone resistance genes identified in *S. enterica* were *qnr*A (33.3%), *qnr*B (20.0%) and *qnr*S (6.7%) while *qnr*A and *qnr*B were detected in 33.3% of *E. coli* isolates. Class 1 integron was detected in 13.3% of *S. enterica* and in 14.3% of *E. coli* isolates. Class 2 integron as well as the colistin resistance gene *mcr*-1 was not found in any of *E. coli* or *S. enterica* isolates.

**Conclusions:**

This study showed high prevalence of *S. enterica* and *E. coli* as foodborne pathogens in raw chicken and beef meat in Nile Delta, Egypt. The emergence of antimicrobial resistance in *S. enterica* and *E. coli* isolates is of public health concern in Egypt. Molecular biological investigation elucidated the presence of genes associated with antibiotic resistance as well as class 1 integron in *S. enterica* and *E. coli*.

## Background

In spite of the improved technology and hygienic practices in developed countries at all stages of poultry and beef meat production, foodborne infections remain as a continuous threat to human and animal health. *Escherichia* (*E*.) *coli* and *S. enterica* serovars are the dominant members of *Enterobacteriaceae* causing foodborne infections. The expansion of antibiotic resistance in bacteria is also an emerging public health hazard due to the compromised efficacy in the treatment of infectious diseases [1].

The continuous exposure of bacterial strains by β-lactams has led to dynamic and massive production and mutation of β-lactamases [2]. Extended-spectrum β-lactamases (ESBLs) are derived from point mutations in the *bla*_TEM-1_ and *bla*_SHV-1_ *β*-lactamase genes, which cannot hydrolyse cephamycins and are inhibited by clavulanic acid [3], while ampicillin class C *β*-lactamase (*Amp*C) enzymes are active on cephamycins as well as oxyiminocephalosporins and monobactams [4]. ESBLs are mostly located on mobile genetic elements (plasmids or integrons), which can facilitate their mobility from bacterial species to others by horizontal gene transfer [5].

In Egypt, *Salmonella* isolates from chicken meat and organs showed high resistance to different antibiotic classes [6–8] where also, ESBL-producing *E*. *coli* were isolated from retail chicken meat and dairy products in Egypt [9, 10].

Quinolones considered as drugs of choice for treatment of human infections caused by Gram-negative bacteria. However, resistance to quinolones has been emerged over the time due to misuse and/or overdose of drugs in human and veterinary practice [11]. Plasmid-mediated quinolone resistance (PMQR) represented by quinolone resistance (*qnr*) genes is widely distributed among bacteria [12–14].

Integrons are genetic elements able to capture individual antibiotic resistance genes including β-lactamases encoding genes and stimulate their transcription and expression [15]. The capture and spread of antibiotic resistance determinants by integrons stimulates the rapid evolution of multidrug resistances (MDR) among Gram-negative bacteria [16]. Among five main integron classes, integrons 1 and 2 are most frequent in Gram-negative bacteria. More than 70 different antibiotic resistance genes have been characterized within integrons [16].

Colistin as antimicrobial substance was used against Gram-negative bacteria. Its usage has been limited due to its systemic toxicity but it was re-introduced as a last-line option in treatment of human infections [17, 18].

This study aimed to discuss the prevalence, serotyping, antibiotic resistance and characterization of resistance-associated genes in *S. enterica* serovars and *E. coli* isolated from beef and poultry meat products in the Nile Delta, Egypt.

## Methods

### Sampling, isolation and identification of bacteria

One hundred and eighty samples were randomly collected aseptically (90 chicken and 90 beef meat and organs) from slaughterhouses and markets in four districts in northern Egypt (Dakahlia, Damietta, Kafr El-Sheikh and Gharbia governorates). Briefly, 30 samples of freshly slaughtered chickens and 30 samples of native frozen chickens from restaurants and supermarkets (each 15 from breast and from thigh muscles and skin) were taken. Thirty samples of organs (gizzard and liver) were collected from freshly slaughtered chickens. In addition, 30 beef meat samples of freshly slaughtered carcasses (neck, brisket, flank and rump muscles) were obtained from slaughterhouses. Thirty samples from locally frozen beef meat, collected from supermarkets and restaurants and 30 samples from organs (liver, spleen and heart) from freshly slaughtered animals were used in this study (Tables 1, 2).

**Table 1:** Sources, number of *S. enterica* isolates and results of their serovar identification

Serovar	Poultry				Beef				(Poultry + beef) n (%)
	Fresh meat (n = 30)	Frozen meat (n = 30)	Fresh organs (n = 30)	Total n (%)	Fresh meat (n = 30)	Frozen meat (n = 30)	Fresh organs (n = 30)	Total n (%)	
*S.* Typhimurium	2	0	1	3 (3.3)	3	1	2	6 (6.7)	9 (60.0)
*S*. Paratyphi A	0	1	1	2 (6.7)	0	0	1	1 (3.3)	3 (20.0)
*S.* Enteritidis	1	0	0	1 (3.3)	1	0	0	1 (3.3)	2 (13.3)
*S.* Kentucky	1	0	0	1 (3.3)	0	0	0	0 (0)	1 (6.7)
Total	4	1	2	7 (7.8)	4	1	3	8 (8.8)	15 (100)

**Table 2:** Sources, number of *E. coli* isolates and results of their serogroup identification

Serogroup	Fresh poultry meat (n = 30)	Frozen poultry meat (n = 30)	Fresh poultry organs (n = 30)	Fresh beef meat (n = 30)	Frozen beef meat (n = 30)	Fresh beef organs (n = 30)	Total number of isolates	% of *E. coli* isolates
O1	1	1	0	0	0	0	2	9.5
O18	2	1	1	0	0	0	4	19
O20	1	–	1	–	–	–	2	9.5
O78	1	0	0	1	1	1	4	19
O103	0	0	0	0	1	0	1	4.8
O119	1	–	–	1	–	–	2	9.5
O126	0	0	1	0	1	0	2	9.5
O145	0	0	1	0	0	0	1	4.8
O146	0	1	0	0	0	0	1	4.8
O158	0	0	0	1	1	0	2	9.5
Total	6	3	4	3	4	1	21	100

Ten grams of each meat and organ sample were incised using sterile scalpel and forceps, transported immediately to the laboratory in ice bags, then transferred into sterile homogenizer flask containing 45 ml of nutrient broth (Oxoid, Manchester, UK). The mixture was allowed to stand for 15 min at room temperature. From each sample, 1 ml was added to 9 ml of buffered peptone water (Oxoid) for *E. coli* cultivation and 9 ml of Rappaport–Vassiliadis broth (Oxoid) for *S. enterica* isolation. After aerobic incubation at 37 °C overnight, a loopful from Rappaport–Vassiliadis broth was streaked on xylose lysine deoxycholate (XLD) agar (Oxoid) and from buffered peptone water on eosin methylene blue (EMB) agar (Oxoid). The inoculated plates were incubated aerobically at 37 °C for 18–24 h. Suspected colonies were identified biochemically using API 20E (bioMérieux, Marcy-l’Étoile, France).

All biochemically confirmed *Salmonella* isolates were serotyped on the basis of somatic (O) and flagellar (H) antigens by slide agglutination test using commercial antisera (SIFIN, Berlin, Germany) following Kauffman–White scheme [19]. Serological identification of *E. coli* was carried out using slide agglutination method using commercial antisera (SIFIN). The serotyping was carried out at the Serology Unit, Animal Health Research Institute, Dokki, Egypt, and the Bacteriology Laboratory, Central Laboratories of Ministry of Health, Cairo, Egypt.

### DNA extraction and purification

The identified bacterial cultures were cultivated in Luria–Bertani (LB) broth (Oxoid) at 37 °C overnight. DNA was extracted and purified using DNeasy Blood and Tissue Kits (Qiagen, Manchester, UK) according to the manufacturer’s instructions.

### Molecular identification of *S. enterica* serovars and *E. coli*

The isolated strains were further identified as *S. enterica* and *E. coli* using PCR assays. The amplification targets and primers (Eurofins, Yokohama, Japan) are listed in Table 3. All phenotypically identified *S. enterica* serovars were tested for harbouring *omp*C gene. Isolated *E. coli* were confirmed by species-specific PCR targeting the 16S *r*RNA genes and resulting in a 585 bp amplicon. PCR assays used in this study resulted in *Salmonella*, *S*. Enteritidis and *S*. Typhimurium specific products of 204, 304 and 401 bp length, respectively.

**Table 3:** PCR primers, their sequences and amplification targets used in this study

Primer	Sequence (5′–3′)	Amplicon size (bp)	Amplification target	References
*E. coli*, *Salmonella enterica* and *Salmonella* serovar identification				
ECO-f	GAC CTC GGT TTA GTT CAC AGA	585	*E. coli*	[67]
ECO-r	CAC ACG CTG ACG CTG ACC A			
OMPCF	ATC GCT GAC TTA TGC AAT CG	204	*Salmonella* genus	[68]
OMPCR	CGG GTT GCG TTA TAG GTC TG			
ENTF	TGT GTT TTA TCT GAT GCA AGA GG	304	*S.* Enteritidis	[68]
ENTR	TGA ACT ACG TTC GTT CTT CTG G			
TYPHF	TTG TTC ACT TTT TAC CCC TGA A	401	*S*. Typhimurium	[68]
TYPHR	CCC TGA CAG CCG TTA GAT ATT			
β-Lactamases				
TEM-F	ATA AAA TTC TTG AAG ACG AAA	1080	*bla*_TEM_	[69]
TEM-R	GAC AGT TAC CAA TGC TTA ATC			
OXA-F	TCA ACT TTC AAG ATC GCA	591	*bla*_OXA_	[69]
OXA-R	GTG TGT TTA GAA TGG TGA			
OXA-F-2	ATT AAG CCC TTT ACC AAA CCA		Whole *bla*_OXA_	[69]
OXA-R-2	AAG GGT TGG GCG ATT TTG CCA			
OXA-23-F	GAT CGG ATT GGA GAA CCA GA	501	*bla*_OXA-23_	[25]
OXA-23-R	ATT TCT GAC CGC ATT TCC AT			
CTX-M-F	CGC TTT GCG ATG TGC AG	550	*bla*_CTX-M_	[69]
CTX-M-R	ACC GCG ATA TCG TTG GT			
CTX-M-F2	CCA GAA TAA GGA ATC CCA TG		Whole *bla*_CTX-M_	[69]
CTX-M-R2	GCC GTC TAA GGC GAT AAA C			
CMY-F	GAC AGC CTC TTT CTC CAC A	1007	*bla*_CMY_	[69]
CMY-R	TGG AAC GAA GGC TAC GTA			
CMY-F2	ACG GAA CTG ATT TCA TGA TG		Whole *bla*_CMY_	[69]
CMY-R2	GAA AGG AGG CCC AAT ATC CT			
SHV-F	TTA TCT CCC TGT TAG CCA CC	795	*bla*_SHV_	[69]
SHV-R	GAT TTG CTG ATT TCG CTC GG			
Integrons				
5′-CS	GGC ATC CAA GCA GCA AG	152	Class 1 integron	[69]
3′-CS	AAG CAG ACT TGA CCT GA			
hep74	CGG GAT CCC GGA CGG CAT GCA CGA TTT GTA	491	Class 2 integron	[69]
hep51	GAT GCC ATC GCA AGT ACG AG			
Plasmid-mediated quinolone resistance gene				
qnrA-F	ATT TCT CAC GCC AGG ATT TG	516	*qnr*A	[69]
qnrA-R	GAT CGG CAA AGG TTA GGT CA			
qnrB-F	GAT CGT GAA AGC CAG AAA GG	469	*qnr*B	[69]
qnrB-R	ACG ATG CCT GGT AGT TGT CC			
qnrS-F	ACG ACA TTC GTC AAC TGC AA	417	*qnr*S	[69]
qnrS-R	TAA ATT GGC ACC CTG TAG GC			
Plasmid-mediated colistin resistance gene				
CLR5-F	CGG TCA GTC CGT TTG TTC	308	*mcr*-1	[24]
CLR5-R	CTT GGT CGG TCT GTA GGG			

The PCR protocol consisted of the following steps: (i) an initial denaturation step of 2 min at 95 °C; (ii) 30 cycles with 1 cycle consisting of 1 min at 95 °C, 1 min at 57 °C, and 2 min at 72 °C; and (iii) a final elongation step of 5 min at 72 °C.

Five-microliter aliquots of reaction mixture were run in electrophoreses using 1.5% agarose gels (Nippon Gene, Tokyo, Japan) and visualized under UV light after ethidium bromide staining.

### Determination of antimicrobial susceptibility profiles

The antimicrobial susceptibility was determined using the Kirby–Bauer disc diffusion test [20]. Briefly, one colony picked up, streaked on Mueller–Hinton blood agar (Oxoid) and incubated at 37 °C overnight. Bacterial colonies were suspended in 0.9% NaCl to obtain a McFarland turbidity of 0.5 (Dr. Lange, photometer CADAS 30, Berlin, Germany) containing 1–2  × 10^8^ colony-forming units (CFU)/ml of *E. coli* strain ATCC 25922. Three hundred μl of the suspension were spread onto the surface of a Mueller–Hinton agar plate (Oxoid) using a sterile swab. The antimicrobial discs (Oxoid) of thirteen clinically used antibiotics, which are used in the Egyptian poultry and cattle farms (Tables 4, 5) were distributed onto the surface of the Mueller–Hinton agar plates using a multi-disc dispenser (Oxoid). The plates were incubated at 37 °C overnight. The diameters of inhibition zones were measured using sliding calipers and interpreted using standard break points according to Clinical and Laboratory Standards Institute [21].

**Table 4:** Breakpoint values of antimicrobial agents according to CLSI, 2011 and phenotypic antimicrobial susceptibility profiles of 15 *S. enterica* isolates used in this study

Antibiotic class	Antimicrobial agent	Conc. (μg)	Zone diameter (mm)			*S.* Typhimurium (9)			*S.* Enteritidis (2)			*S.* Kentucky (1)			*S.* Paratyphi A (3)			Total *Salmonella* (15)		
			R	I	S	R	I	S	R	I	S	R	I	S	R	I	S	R	I	S
Penicillin	Amoxycillin–clavulanic acid	20	≤ 13	14–17	≥ 18	5	4	–	–	2	–	1	–	–	2	1	–	8 (53%)	7 (47%)	–
	Ampicillin	10	≤ 13	14–16	≥ 17	7	2	–	2	–	–	1	–	–	3	–	–	13 (87%)	2 (13%)	–
Cephalosporin	Cefotaxime	30	≤ 22	23–25	≥ 26	7	1	1	2	–	–	–	1	–	3	–	–	12 (80%)	2 (13%)	1 (7%)
	Cefpodoxime	20	≤ 17	18–20	≥ 21	7	2	–	1	1	–	–	1	–	1	2	–	9 (60%)	6 (40%)	–
	Ceftazidime	30	≤ 17	18–20	≥ 21	3	5	1	–	2	–	–	1	–	1	2	–	4 (26%)	10 (67%)	1 (7%)
	Ceftriaxone	30	≤ 19	20–22	≥ 23	3	6	–	–	2	–	–	1	–	2	1	–	5 (33%)	10 (67%)	–
Miscellaneous	Chloramphenicol	30	≤ 12	13–17	≥ 18	–	5	4	–	–	2	–	1	–	–	1	2	–	7 (47%)	8 (53%)
	Colistin	10	≤ 11	12–13	≥ 14	–	–	9	–	–	2	–	–	1	–	–	3	–	–	15 (100%)
Fluoroquinolone	Ciprofloxacin	5	≤ 15	16–20	≥ 21	–	3	6	–	1	1	–	1	–	–	–	3	–	5 (33%)	10 (67%)
	Enrofloxacin	5	≤ 17	18–20	≥ 21	1	7	1	–	1	1	–	1	–	–	2	1	1 (7%)	11 (73%)	3 (20%)
	Nalidixic acid	30	≤ 13	14–18	≥ 19	3	6	–	1	1	–	1	–	–	–	3	–	5 (33%)	10 (67%)	–
Aminoglycoside	Streptomycin	10	≤ 11	12–14	≥ 15	2	7	–	1	1	–	1	–	–	–	2	1	4 (26%)	10 (67%)	1 (7%)
Tetracycline	Tetracycline	30	≤ 11	12–14	≥ 15	3	6	–	2	–	–	1	–	–	–	3	–	6 (40%)	9 (60%)	–
Sulphonamide	Trimethoprim/sulphamethoxazole	25	≤ 4	3	≥ 2	4	2	3	1	1	–	1	–	–	2	1	–	8 (53%)	4 (26%)	3 (20%)

*S* sensitive, *I* intermediate, *R* resistant

**Table 5:** Breakpoint values of antimicrobial agents according to CLSI, 2011 and phenotypic antimicrobial susceptibility profiles of *E. coli* isolates used in this study

Antibiotic class	Antimicrobial agent	Conc. (μg)	Zone diameter (9)			*E. coli* (21)									Resistance rate		
						Poultry (15)			Beef (6)			Total (21)			Poultry	Beef	Total
			R	I	S	R	I	S	R	I	S	R	I	S	%	%	%
Penicillin	Amoxycillin–clavulanic acid	20	≤ 13	14–17	≥ 18	10	4	1	3	3	–	13	7	1	66.7	50.0	61.9
	Ampicillin	10	≤ 13	14–16	≥ 17	12	2	1	3	3	–	15	5	1	80.0	50.0	71.4
Cephalosporin	Cefotaxime	30	≤ 22	23–25	≥ 26	6	7	2	1	3	2	7	10	4	40.0	16.7	33.3
	Cefpodoxime	20	≤ 17	18–20	≥ 21	3	12	–	2	4	–	5	16	–	20.0	33.3	23.8
	Ceftazidime	30	≤ 17	18–20	≥ 21	5	9	1	–	6	–	5	15	1	33.3	0	23.8
	Ceftriaxone	30	≤ 19	20–22	≥ 23	3	5	7	1	3	2	4	8	9	20.0	16.7	19.0
Miscellaneous	Chloramphenicol	30	≤ 12	13–17	≥ 18	3	9	3	1	1	4	4	10	7	20.0	16.7	19.0
	Colistin	10	≤ 11	12–13	≥ 14	–	1	14	–	–	6	–	1	20	–	–	–
Fluoroquinolone	Ciprofloxacin	5	≤ 15	16–20	≥ 21	4	3	8	2	2	2	6	5	10	26.7	33.3	28.6
	Enrofloxacin	5	≤ 17	18–20	≥ 21	2	5	8	1	4	1	3	9	9	13.3	16.7	14.3
	Nalidixic acid	30	≤ 13	14–18	≥ 19	5	12	3	1	2	3	6	14	6	33.3	16.7	28.6
Aminoglycoside	Streptomycin	10	≤ 11	12–14	≥ 15	9	5	1	4	2	–	13	7	1	60.0	66.7	61.9
Tetracycline	Tetracycline	30	≤ 11	12–14	≥ 15	12	3	–	5	1	–	17	4	–	80.0	83.3	81.0
Sulphonamide	Trimethoprim/sulphamethoxazole	25	≤ 4	3	≥ 2	10	2	3	3	–	3	17	2	6	66.7	50.0	81.0

*S* sensitive, *I* intermediate, *R* resistant

The antimicrobial susceptibility of colistin was determined by disc diffusion susceptibility testing using colistin discs (Oxoid) containing 10 μg. The disc zone diameters was interpreted according to previous report [22].

### Molecular detection of antimicrobial resistance associated genes

*Escherichia coli* and *S. enterica* isolates were tested for β-lactamase-encoding genes *bla*_TEM_, *bla*_SHV_, *bla*_CTX-M_, *bla*_OXA_ and *bla*_CMY_ by PCR using universal primers for the corresponding gene families (Table 3) as described previously [23]. PCR amplification was also used for screening of plasmid-mediated quinolone resistance genes *qnr*A, *qnr*B and *qnr*S.

For detection of variants of *bla*_TEM_, *bla*_SHV_, *bla*_CTX-M_, *bla*_OXA_ and *bla*_CMY_ amplified PCR fragments were purified from agarose gels using Nucleospin Gel Extraction Kit (Macherey–Nagel, Düren, Germany). The products were sequenced and the sequencing results were analyzed using BLAST (http://blast.ncbi.nlm.nih.gov/blast.cgi).

Colistin resistance associated gene *mcr*-1was identified using a PCR assay (Table 3) as described by Liu et al. [24]. PCR for detection of carbapenemases *bla*OXA-23 gene was performed using OXA-23-F and OXA-23-R (Table 3). PCR reaction and conditions were performed according to Braun et al. [25].

### Detection and sequencing of class 1 and class 2 integrons

PCR assays for detection of class 1 and class 2 integrons were performed using primers given in Table 3 and yielded PCR fragments that were purified from agarose gels using Nucleospin Gel Extraction Kit (Macherey–Nagel, Düren, Germany). The products were sequenced in Genome Centre, Gifu University, Japan. The sequencing results were analyzed using BLAST (http://blast.ncbi.nlm.nih.gov/blast.cgi).

## Results

Out of 180 examined samples 15 *S. enterica* (8.3%) (7 from poultry and 8 from beef) (Table 1) as well as 21 *E. coli* (11.7%) (15 from poultry and 6 from beef) (Table 2) were isolated and identified using bacteriological methods and biochemical characterization.

### Serological characterization of *S. enterica* and *E. coli*

Fifteen isolated *S. enterica* were typed as 9 *S. enterica* serovar Typhimurium (60.0%; 3 from chicken and 6 from beef), 3 *S. enterica* Paratyphi A (20.0%; 2 from chickens and 1 from beef), 2 *S. enterica* serovar Enteritidis (13.3%; 1 from chicken and 1 from beef) and 1 *S. enterica* serovar Kentucky (6.7%) from chicken sample (Table 1).

Twenty-one *E. coli* isolates were characterized as 4 O18 (19.0%) and 4 O78 (19.0%) serotypes. Other serotypes are given in Table 2.

### Molecular identification of *Salmonella* serovars

All 15 recovered *S. enterica* serovars harboured *omp*C gene, which confirms *Salmonella* genus. Nine *S. enterica* serovar Typhimurium (60.0%) and two *S. enterica* serovar Enteritidis (13.3%) were identified by means of serovar-specific bands using PCR (Table 3). Four other isolates could not be typed with PCR method (26.7%).

### Determination of antimicrobial susceptibility profiles

The results of determination of antimicrobial resistance of *S. enterica* and *E. coli* isolates to thirteen antibiotics are given in Tables 4 and 5. Most of the *S. enterica* isolates showed resistance to ampicillin (87.0%) and cefotaxime (80.0%) and all were susceptible to chloramphenicol, colistin and ciprofloxacin. Resistance to other antibiotics was shown in Table 4.

Seven isolates of *S. enterica* serovar Typhimurium were resistant to ampicillin, cefotaxime and cefpodoxime (77.8%), while 6 isolates (66.7%) were highly sensitive to ciprofloxacin. Resistance to other antibiotics was weaker developed.

Both *S. enterica* serovar Enteritidis isolates were resistant to ampicillin, cefotaxime and tetracycline. Additionally, one isolate showed resistance to streptomycin, nalidixic acid, trimethoprim/sulphamethoxazole and streptomycin.

In addition to the entire resistance to ampicillin and cefotaxime, majority of the *S. enterica* serovar Paratyphi A isolates were resistant to amoxicillin–clavulanic acid, ceftriaxone and trimethoprim/sulphamethoxazole.

*Salmonella enterica* serovar Kentucky isolate showed no pronounced susceptibility to any of the tested antibiotics and was resistant to six of them (Table 4). Seven out of 15 *S. enterica* serovars (46.7%) revealed phenotypic multidrug resistance exhibiting by resistance to three or more classes of antibiotics (Table 4).

*Escherichia coli* isolates showed resistance to tetracycline, ampicillin, streptomycin, trimethoprim/sulphamethoxazole and amoxicillin–clavulanic acid with 80.9, 71.4, 61.9, 61.9 and 61.9%, respectively. Ten (47.6%), 9 (42.8%) and 9 (42.8%) isolates were susceptible to ciprofloxacin, enrofloxacin and ceftriaxone, respectively. All tested isolates were susceptible to colistin. Thirteen *E. coli* isolates (61.9%) were characterized as multidrug resistant (Table 5).

The multidrug resistance of isolated *Salmonella* and *E. coli* was shown in Tables 6 and 7, respectively.

**Table 6:** Phenotypic resistance and resistance determinants found in *S. enterica* isolates in this study

Source	Serovar	Resistance phenotype	Resistance genes/class 1 integrons
Poultry			
Fresh poultry meat	Typhimurium	CPD, CTX, NAL	*bla*_CTX-M-1_, *qnr*A, *qnr*B, *qnr*S
Fresh poultry meat	Typhimurium	AMC, AMP, CPD	*bla*_TEM-1_
Fresh poultry meat	Enteritidis	AMP, CTX, TET	*bla*_CTX-M-1_, *bla*_TEM-1_
Fresh poultry meat	Kentucky	AMC, AMP, NAL, STR, TET, T/S	*bla*_TEM-1_, *qnr*A
Fresh poultry organs (liver)	Typhimurium	AMC, AMP, CPD, CRO, CTX, NAL, T/S	*bla*_CTX-M-1_, *bla*_TEM-1_, *qnr*A, *dfrA15*, *dfrA17*
Fresh poultry organs (liver)	Paratyphi A	AMC, AMP, CAZ CRO, CTX, T/S	*bla*_CTX-M-14_, *bla*_TEM-1_
Frozen poultry meat	Paratyphi A	AMC, AMP, CRO, CTX, T/S	*bla*_CTX-M-14_, *bla*_TEM-1_
Beef			
Fresh beef meat	Typhimurium	AMP, CAZ, CTX	*bla*_CTX-M-1_
Fresh beef meat	Typhimurium	AMP, CAZ, CRO, CTX	*bla*_CTX-M-1_
Fresh beef meat	Typhimurium	AMC, CPD, CTX, TET, T/S	*bla*_CTX-M-3_, *bla*_TEM-1_
Fresh beef meat	Enteritidis	AMP, CPD, CTX, NAL, STR, TET, T/S	*bla*_CMY-2_, *bla*_CTX-M-1_, *bla*_TEM-1_, *qnr*A, *qnr*B
Fresh beef organs (spleen)	Typhimurium	AMP, CPD, CTX, STR,TET, T/S	*bla*_CTX-M-14_, *bla*_TEM-1_
Fresh beef organs (liver)	Typhimurium	AMC, AMP, CPD	–
Fresh beef organs (liver)	Paratyphi A	AMP, CPD, CTX	*bla*_TEM1_
Frozen beef meat	Typhimurium	AMC, AMP, CAZ, CPD, CRO, CTX, ENR, NAL, STR, TET, T/S	*bla*_CMY-2_, *bla*_CTX-M-1_, *bla*_TEM1_, *qnr*A, *qnr*B, *aadA2*

*AMC* amoxycillin–clavulanic acid, *AMP* ampicillin, *CAZ* ceftazidime, *CPD* cefpodoxime, *CRO* chloramphenicol, *CTX* cefotaxime, *ENR* enrofloxacin, *NAL* nalidixic acid, *STR* streptomycin, *TET* tetracycline, *T/S* trimethoprim/sulphamethoxazole

**Table 7:** Phenotypic resistance and resistance determinants found in *E. coli* isolates in this study

Source	*E. coli* serotype	Resistance phenotype	Resistance genes/class 1 integron
Poultry			
Fresh poultry meat	O1	STR, TET	–
Fresh poultry meat	O18	AMP, CIP, CPD, NAL, STR, TET, T/S	*qnr*A, *qnr*B
Fresh poultry meat	O18	AMC, AMP, CHL, CIP, CRO, CTX, NAL, STR, TET, T/S	*bla*_CTX-M-1_, *bla*_OXA-1_, *qnr*A, *qnr*B
Fresh poultry meat	O78	AMC, AMP, CAZ, TET, T/S	*bla*_CTX-M-1_, *bla*_TEM-1_
Fresh poultry meat	O119	AMC, AMP, CAZ, CTX, STR, TET, T/S	*bla*_CTX-M-14_, *bla*_TEM-104_
Fresh poultry meat	O20	AMC, AMP, CPD, TET, T/S	*bla*_CTX-M1_, *bla*_OXA-1_, *bla*_TEM-1_
Fresh poultry organs (liver)	O18	AMC, AMP, CAZ, CHL, CIP, CRO, CTX, ENR, NAL, STR, TET, T/S	*bla*_CTX-M-1_, *bla*_TEM-104_,*qnr*A, *qnr*B, *dfr*A1-orf
Fresh poultry organs (liver)	O126	AMP, CTX, STR	*bla*_CTX-M-1_
Fresh poultry organs (gizzard)	O20	AMC, AMP, STR, T/S	*bla*_TEM-1_
Fresh poultry organs (liver)	O145	CAZ, TET	–
Frozen poultry meat	O1	CTX, NAL, TET	*qnr*A, *qnr*B
Frozen poultry meat	O18	AMC, AMP, STR, TET, T/S	*bla*_OXA-1_, *bla*_TEM-1_
Frozen poultry	078	AMC, AMP, CPD	
Frozen poultry meat	O146	AMC, AMP, TET, T/S	*bla*_TEM-1_
Frozen organ poultry	078	AMC, AMP, CAZ, CHL, CIP, CRO, CTX, ENR, NAL, STR, TET, T/S	*bla*_CTX-M-14_,*bla*_TEM-1_, *qnr*A, *qnr*B, *dfr*A15
Beef			
Fresh beef meat	O119	CPD	–
Fresh beef meat	O158	CPD, TET	–
Fresh beef organs (liver)	O78	AMC, AMP, CHL, CIP, CRO, CTX, ENR, NAL, STR, TET, T/S	*bla*_CTX-M-1_, *bla*_TEM-104_, *qnr*A*, qnr*B, *dfr*A1, *dfr*A1-orf
Frozen beef meat	O103	STR, TET	*bla*_CTX-M-1_
Frozen beef meat	O126	AMC, AMP, CIP, STR, TET, T/S	*bla*_TEM-1_, *qnr*A, *qnr*B
Frozen beef meat	O158	AMC, AMP, STR, TET, T/S	*bla*_TEM-1_

*AMC* amoxycillin–clavulanic acid, *AMP* ampicillin, *CAZ* ceftazidime, *CPD* cefpodoxime, *CRO* chloramphenicol, *CTX* cefotaxime, *ENR* enrofloxacin, *NAL* nalidixic acid, *STR* streptomycin, *TET* tetracycline, *T/S* trimethoprim/sulphamethoxazole

### Molecular detection of resistance-associated genes

Six of 8 screened resistance-associated genes were detected in *S. enterica* serovars by PCR (Table 6). Eleven (73.3%) isolates harboured *bla*_CTX-M_ gene (1, 3 and 14) associated with cefotaxime resistance and 11 (73.3%) isolates harboured *bla*_TEM-1_ gene associated with penicillin and narrow spectrum β-lactamase resistance. Five (33.3%) isolates harboured quinolone resistance gene A (*qnr*A), while 3 (20.0%), 2 (13.3%) and 1 (6.7%) of *S. enterica* serovars possessed *qnr*B, *bla*_CMY_ and *qnr*S genes, respectively. *bla*_OXA_ associated with resistance to ampicillin and cephalothin and *bla*_SHV_ associated with plasmid-mediated ampicillin resistance were not found in any isolate.

In *E. coli* isolates, 5 of 8 screened resistance-associated genes were detected by PCR (Table 7). Eleven (52.4%) out of 21 isolates harboured *bla*_TEM_ (1 and 104), while *bla*_CTX-M_ (1 and 14) was detected in 9 isolates (42.9%). Seven isolates (33.3%) carried *qnr*A and *qnr*B and in three isolates (14.3%) *bla*_OXA-1_ was detected. Genes *bla*_CMY_, *bla*_SHV_ and *qnr*S were not detected in any of the *E. coli* isolates.

Plasmid-mediated colistin resistance gene *mcr*-1 and carbapenemase resistance gene *bla*OXA-23 were not identified neither in *S. enterica* nor in *E. coli* isolates using PCR assay.

### Integron gene cassettes and DNA sequencing

Class 1 integron was detected in two isolates of *S.* Typhimurium using PCR (Table 6). The inserted gene cassettes identified three types of antimicrobial resistance genes associated with class 1 integron: dihydrofolate reductase types (*dfr*A15 and *dfr*A17) which confer resistance to trimethoprim, and aminoglycoside adenyltransferase type *aad*A2 that confers resistance to streptomycin and spectinomycin.

Three *E. coli* isolates of serotypes O18, O78 and O78 were harbouring class 1 integron (Table 7). The inserted gene cassettes identified three types of class 1 integron. The identified antimicrobial resistance genes were dihydrofolate reductase types; *dfr*A1, *dfr*A15 and *dfr*A1-orf which confer resistance to trimethoprim.

All *S. enterica* and *E. coli* isolates were negative for class 2 integrons.

## Discussion

Salmonellosis and *Salmonella* infections considered as critical threats to human and animal health. In this study, the prevalence of *S. enterica* serovars in chicken and beef meat was 8.3%, which was considerably lower than incidence rates that reported in Ethiopia (12.0% in raw meat) [26], Canary islands (16.5% in chicken meat) [27], Northwestern Spain (17.9% in chicken) [28], Ethiopia (17.9% in chicken and giblets) [29], and Egypt (10.0% in poultry meat) [7]. On the other hand, it was higher than reported previously in meat products in Egypt (6.6%) and in ground beef in the United States (4.2%) [30, 31].

In total, *S. enterica* serovar Typhimurium was the dominant serovar. These results were in accordance with previous study from chicken products in Cambodia and Thailand [32]. Controversially, *S*. *enterica* serovar Enteritidis was the dominant serotype in imported frozen poultry samples from Brazil to Canary Islands and in chicken carcasses in Spain [27, 28]. *Salmonella enterica* serovar Kentucky was frequently detected in samples coming from US to Canary Islands [27].

Prevalence of *E. coli* in chicken meat and organs was 16.7% which was lower than in Nigeria (43.4%) in frozen poultry meat [33] but higher than in Korea (4.9% in poultry meat) [34]. Prevalence of *E. coli* in beef meat was 6.7%, while in Korea and Iran were found 4.1 and 29.0%, respectively [34, 35].

In this study, 19.0% of *E. coli* isolates typed as O78 was mainly from poultry products, while in China, O78 was identified in 60.0% of *E. coli* isolated from chicken and ducks [36]. *E. coli* type O158 identified in 9.5% only in beef meat while O158 detected in 22.7% of food isolates in Chile [37].

Bacterial antimicrobial resistance is a global emerging problem of public health concern.

In the current study, a high percentage of *S. enterica* serovars were resistant to ampicillin and cefotaxime. The resistance to other antimicrobial agents was variable while all isolates were sensitive to chloramphenicol and ciprofloxacin. The results were in a partial accordance with results of previous reports stating that all *S. enterica* isolated from chicken meat and beef were sensitive to ciprofloxacin [38]. 72.4% of *Salmonella* isolates in Thai meat products were resistant to ampicillin while 71.0% of isolates in Cambodian meat products were resistant to sulfamethoxazole [32]. Controversially, *S. enterica* isolated from animals and food of animal origin in Italy were highly resistant to ampicillin, chloramphenicol, streptomycin and tetracycline [39] and all *Salmonella* isolated from beef in Tunisia were susceptible to amoxicillin and clavulanic acid [40].

In total, 46.7% of *S. enterica* serovars showed multidrug resistance, which was higher than resistance of *Salmonella* isolated from raw chicken (31.0%) in Pennsylvania during [41]. Less multidrug resistant *S. enterica* isolates (6.0%) were identified in cattle hides and carcasses in the US [42].

The results of this study showed that the antimicrobial resistance of *E. coli* isolated from poultry was higher than from beef to the most of tested antibiotics. The high resistance to tetracycline, ampicillin, amoxycillin–clavulanic acid, trimethoprim/sulphamethoxazole and streptomycin in this study was in agreement with previous reports conducted in Egypt [10, 43] and Algeria [44]. While in Spain, most of *E. coli* isolated from diarrhoeic and healthy lambs were highly resistant to tetracycline and streptomycin but show lower resistance to ampicillin [45]. Most of *E. coli* isolates in this study were sensitive to enrofloxacin, chloramphenicol and ceftriaxone which is in agreement with previous results in Spain [45].

In this study, 61.9% of *E. coli* isolates were multidrug resistant. Similar results reported previously in Ghana and US [23, 46].

Most of the phenotypically antibiotic resistant *S. enterica* serovars carried antibiotic resistance marker genes associated with β-lactams and quinolones.

The β-lactamase encoding gene *bla*_TEM_ conferring resistance to penicillins and first-generation cephalosporins was detected in 73.3% of *S. enterica* serovars and in 76.9% of ampicillin-resistant isolates which is significantly high in comparison to 57.3% (*bla*_TEM-1_) in *S. enterica* isolated from retail chickens in China [47]. Another β-lactamase encoding gene *bla*_CMY-2_, an AmpC beta-lactamase gene that confers resistance to a wide variety of β-lactam antibiotics detected in 13.3% of isolates. In contrast, in 4.7% of *S. enterica* serovars Typhimurium and Enteritidis originated from diseased broilers in Egypt *bla*_CMY-2_ was identified [48] and all resistant *S. enterica* serovar Typhimurium isolated from retail meat in US were harbouring *bla*_CMY_ [49]. The same gene could not be detected previously in any *S. enterica* serovar Typhimurium isolated from chicken meat in Egypt [8].

*bla*_CTX-M_ could be identified in 73.3% of isolated *S.* enterica serovars. In 24.0 and 18.8% of different *Salmonella* serovars isolated from retail chicken carcasses in China, *bla*_CTX-M-15_ and *bla*_CTX-M-3_ were detected, respectively [47].

The *bla*_SHV_ is responsible for the plasmid-mediated ampicillin resistance and β-lactamase encoding genes *bla*_OXA_ conferring resistance to ampicillin and cephalothin not detected in any of *S. enterica* serovars in this study. This result was in accordance with a result for *S. enterica* serovar Typhimurium isolated from chicken meat in Egypt [8]. In contrast, 30.2% of ESBL-producing *Salmonella* isolated from raw chicken carcasses in China were harbouring *bla*_OXA-1_ [47].

Although none of *S. enterica* serovars were phenotypically resistant to ciprofloxacin*, qnr*A, *qnr*B and *qnr*S genes were detected in 33.3, 20.0 and 6.7%, respectively in all *S. enterica* serovars and in 100, 60.0 and 20.0%, respectively in *S. enterica* resistant to nalidixic acid. A different result showed that 1.16% of nalidixic acid-resistant *S. enterica* serovars isolated from animal products in Tunisia carried *qnr* gene [50]. In other studies in China *qnr*A, *qnr*B and *qnr*S genes detected with low percentage in *S. enterica* serovar Enteritidis isolated from retail raw poultry [51, 52].

The *bla*_TEM_ gene was detected in 52.3% of all isolated *E. coli* and in 73.3% of ampicillin-resistant isolates. Other studies detected *bla*_TEM_ in 97.1 and 75.0% of *E. coli* isolates from lambs in Spain and meat products in Cambodia, respectively [32, 45]. In China, *bla*_TEM_ was identified in 7.8% of ESBL-producing *E. coli* recovered from meat products [53].

The β-lactamase encoding gene *bla*_CMY_ was not found in any of the isolated *E. coli*. In contrast, *bla*_CMY-2_ was detected in 89.0% *E. coli* isolated from poultry meat in Denmark [54] and in 12.5% of ESBL-producing *E. coli* isolated from meat products in Cambodia [32].

The *bla*_CTX-M_ detected in 42.9% of *E. coli* isolated in this study. In Ghana 50.0% of ESBL-producing *E. coli* isolates from meat harboured *bla*_CTX-M_ [46] while these β-lactamase encoding genes *bla*_CTX-M-15 and_ *bla*_CTX-M-9_ were detected rarely (1.6%) in *E. coli* isolates from meat products in China [53].

The *bla*_SHV_ gene was not found in any of the *E. coli* isolates. Controversially, previous studies detected *bla*_SHV_ in 9.4, 5.3 and 2.0% in meat products in China, broiler chickens and chicken carcasses in Iran, respectively [53, 55, 56].

*bla*_OXA-1_ was detected in 14.3% of the *E. coli* isolates which is in contrast with previous report conducted in retail meat in US where the gene could not be identified [23].

The most common carbapenemase types were *bla*OXA-23 and *bla*OXA-48 accounting for 47% of all identified carbapenemase genes [25, 57]. In this study, *bla*OXA-23 was not identified in all isolated strains.

In isolated *E. coli*, *qnr*A and *qnr*B detected in 33.3% of isolates, all nalidixic acid and/or ciprofloxacin resistant isolates harboured both genes. While *qnr*B and *qnr*S were identified in 10.4% of nalidixic acid resistant *E. coli* isolated from Algerian retail chicken meat [44] and in 10.0% of *E. coli* isolates obtained from bovine carcasses in Mexico [58]. In contrast, *qnr*A, *qrn*B and *qrn*S genes could not be identified in enrofloxacin-resistant *E. coli* strains from poultry in Mexico [59].

The presence of genetic elements such as integrons and transposons are often associated with multi-resistant phenotypes among *Salmonella* isolates [60]. In this study, class 1 integron detected in 13.3% of *S. enterica* serovars. The identified gene cassettes were *dfr*A15, *dfr*A17 and *aad*A2. In previous studies, class 1 integron identified in 90.0% of multi-drug resistant *Salmonella* isolates from retail chicken meat in Japan and the identified genes cassettes were *dfr*A1, *dfr*A7, *aad*A1, *aad*B, and *cat*B3 [61, 62]. The gene cassettes of class 1 integron which detected in *Salmonella* spp. isolated from poultry meat in Egypt were harbouring *aac* (3)-Id, *aad*A2, *aad*A4, *aad*A7, *sat*, *dfr*A15, *lnu*F and *est*X resistance genes [7]. In a study conducted in Portugal, 75.0% of *S. enterica* isolated from different sources including food products had one or two class 1 integrons [63].

The identified gene cassettes of class 1 integron *dfr*A1, *dfr*A15 and *dfr*A1-orf which confer resistance to trimethoprim were identified in 14.3% of *E. coli* isolated in this study. This is in agreement with *E. coli* isolates from retail chickens in Japan [62] and in 1.9 and 11.4% of isolates from Thai and Cambodian meat samples obtained from slaughterhouses and fresh markets and the most common gene cassette was *dfr*A12-*aad*A2 [32].

Class 2 integrons were not detected in all isolates of this study which is in contrast to 5.6% positive samples in Egypt [10].

In this study, *mcr*-1 gene associated with colistin resistance was neither detected in *S. enterica* nor *E. coli* isolates. This result was in agreement with the result reported by Doumith et al. [64] who investigated 24,000 isolates of *Enterobacteriaceae* from food and humans including *S. enterica* and *E. coli* and found only 15 *mcr*-1 positive isolates. Recently, Quesada et al. identified the gene *mcr*-*1* in nine *S. enterica* and *E. coli* isolates from poultry and swine for the first time in Spain [65]. Jayol et al. found the *mcr*-1 in 13% of *E. coli* and *S. enterica* [66]. Considering the frequent use of colistin in animal production and the importance of this antimicrobial for the control of multi-resistant Gram-negative nosocomial infections in humans, monitoring the dissemination of resistance to colistin is mandatory.

In conclusion, the results of this study showed high prevalence of *S. enterica* and *E. coli* as foodborne pathogens isolated from poultry and beef meat in Egypt. The emergence of antimicrobial resistance of *S. enterica* and *E. coli* isolates is of public concerns in Egypt. Significant resistance was detected to penicillin, cephalosporins, tetracycline and sulphonamides. Dissemination of ESBL and AmpC-β-lactamase resistance-associated genes in *S. enterica* and *E. coli* was determined. Presence of class 1 integron in *S. enterica* and *E. coli* and genes associated with antibiotic resistance was also confirmed. Class 2 integron was not detected in any isolate. Further work should be performed to characterize *S. enterica* and *E. coli* isolates of animal and human origin from the same region sharing the same resistance markers in order to highlight potential horizontal gene transfer by these zoonotic organisms.
